# Equity analysis of Chinese physician allocation based on Gini coefficient and Theil index

**DOI:** 10.1186/s12913-021-06348-w

**Published:** 2021-05-12

**Authors:** Huimin Yu, Shuangyan Yu, Da He, Yuanan Lu

**Affiliations:** 1 School of Economics and Management, Jiangxi University of Traditional Chinese Medicine, Nanchang, 330000 Jiangxi China; 2 Shanghai Health Development Research Center, Shanghai, 200040 China; 3 Department of Public Health Sciences, University of Hawaii at Manoa, Honolulu, HI 96822 USA

**Keywords:** Physician, Fairness, Resource allocation, Lorenz curve (LC), Gini coefficient (G), Theil index (T)

## Abstract

**Background:**

Unequal allocation of medical physician resource represents one of major problems in the current medical service management in China and many other countries. This study is designed to analyze the current distribution of physicians in 31 provincial administrative regions in China, to estimate the fairness of the distribution of physicians and provide a theoretical basis for the improvement of the allocation of physicians.

**Methods:**

This study took physicians from 31 provincial administrative regions in China as the study objects, and the data were obtained from the China Health Statistics Yearbook 2019 and the official website of the National Bureau of Statistics of China. Calculation of the Gini coefficient (G) and the Theil index (T) were carried out by drawing the Lorenz curve. The fairness of present physician location in 31 provincial administrative regions in China was analyzed from the perspective of distribution by both population and service area.

**Results:**

The Gini coefficients of medical physicians in China are 0.003 and 0.88 by population and by service area, respectively. This shows that the distribution of medical physicians is fair basing on population, and there is little difference in the number of physicians per 1000 population in different regions. However, the physician distribution basing on service area is highly unfair and shows a large gap in the number of physicians per square kilometer between different regions. In general, Beijing, Zhejiang, Shanghai, Jiangsu, Shandong, and Tianjin are higher than the overall level of 31 provincial administrative regions. In addition, the number of medical physicians in Zhejiang, Shandong, Beijing and Jiangsu is over-provisioned.

**Conclusion:**

Bridging the number of medical physicians in different regions is a key step to improve the equity of physicians’ resource allocation. Thus, findings from this study emphasize the need to take more measures to reduce physician quality differences between regions, balance and coordinate medical resources. This will increase the access of all citizens to quality medical services.

## Background

As the aging population accelerates and the spectrum of diseases changes, the demand for medical services in China has surged. At the same time, due to the continuous occurrence of injuries and medical incidents, the safety of medical practice environment and other factors have caused the continuous losses of physician resources (PR) [1]. In the shortage of Chinese PR, the fair and effective distribution of medical resources has attracted more and more attention. Due to the desire for improved fairness of the distribution of human resources for health [2], the issue of equality of human resources for health also frequently appears in China's policy development agenda.

According to the law of the People’s Republic of China on Practicing Physicians, medical students who have obtained the qualifications of practicing physicians or practicing assistant physicians must apply for registration with the health administrative department of local government at or above the county level, and then start to engage in the corresponding medical, prevention, and health care businesses. Thus, physicians must have both professional titles and qualifications for practicing medicine. In March 2015, the General Office of the State Council issued the “Notice on Printing and Distributing the Outline of the National Medical Service System Planning (2015-2020)” (Guobanfa [2015] No. 14), which was clearly planned that by 2020, the number of practicing (assistant) physicians per 1000 permanent residents in China will reach 2.5. During 2018–2019, the State Council successively issued the Opinions on Promoting the Development of “Internet + Medical and Health” (Guobanfa [2018] No. 26), and the “Notice on Printing and Distributing Key Tasks for Deepening the Medical and Health System in 2019” (National Banfa [2019] No. 28) and other policies. The purpose is to adopt measures such as the adoption of Internet technology, the construction of national medical centers and regional medical centers, This will speed up the implementation of medical resources, and improve the level of medical services in areas where quality medical resources are in short supply.

With a population of more than 1.4 billion, China has a large demand for physician resources. Especially in the case of the outbreak of the COVID-19 epidemic in China, sufficient PR become even more crucially important. There are a total of 34 provincial administrative regions in China. Of which, Taiwan, Hong Kong, and Macau implement their local-specific medical and health systems. This study mainly focuses on the physicians in the rest 31 regions of China who implement the same medical and health systems with the analysis on the current situation of the fairness of the allocation of physicians 'resources in China. Findings from this study will provide a theoretical basis for China and other regions to take effective measures to optimize the allocation of physicians' resources.

The issue of fairness in the allocation of medical and health resources has become a key research topic in the public health field in many countries [3–5]. In the early days, the polarization of China's medical and health services was serious. In 2000, the World Health Organization evaluated and ranked the fairness of health financing and distribution of its 191-member countries, and China ranked 188^th^ [6]. The unreasonable distribution of medical resources and low fairness in China have attracted more and more attention. Numerous studies have shown that inequality in the distribution of PR can be determined by many factors including: 1) Economic development will have a significant impact on the distribution of human resources [7, 8]; 2) Population density has also been identified as a factor causing unequal distribution of human resources for health [9], and 3) There are also government health expenditures [10].

Unequal allocation of medical physician resource is one of well-known problems for the present management of medical resources and services in many countries, including China. In this study, the fairness of the current distribution of physicians in 31 provincial administrative regions in China was analyzed by using Gini coefficient (G) and Theil index (T) methods. Findings from this study may provide a theoretical basis useful for the improved management of the allocation of physicians in future.

## Methods

### Data collection

As of the end of 2018, the total number of practicing (assistant) physicians in China was approximately 3,607,156, the average number of physicians per 1,000 population in the 31 provincial administrative regions is 2.62. The data for this study came from two sources: a). China Health Statistics Yearbook 2019, and b). China National Bureau of Statistics official website [11]. Based on the statistical description of Chinese practicing (assistant) physicians in 2018, the basic information of Chinese physicians was analyzed. Basing on the allocation of PR in the 31 provincial regions in China, fairness analysis and evaluation were performed using Lorenz curve (LC) and Gini coefficient.

### Fairness assessment

The Lorenz curve is used to evaluate the fairness of the allocation of medical and health resources in the field of public health. The basic principle is that income or resources are divided into several levels according to different populations or regions, and they are accumulated according to the percentage from small to large, and are represented by the vertical axis; The corresponding percentage of the population remains the same, and is expressed on the horizontal axis after accumulation. The Lorenz curve is then generated by connecting the corresponding points. If the curve is closer to the ideal fair line, this means the smaller the income gap or the closer the distribution of resources is to equity. Conversely, the farther the curve is from the absolute fairness line, the worse the fairness is.

Gini coefficient (G) or Gini index is a statistical index reflecting fairness calculated based on the LC with various calculation methods. The general method for calculating the G of medical and health resource allocation in China is to directly apply the Lorenz curve formed by the cumulative statistical points, and then calculate the G according to the trapezoidal area formed by each segment.

The specific calculation formula of Gini coefficient is.

$$ \mathrm{G}=\sum \limits_{i=1}^n\left({\mathrm{X}}_{i+1}-{X}_i\right)\left(Y{}_{i+1}+{Y}_i\right) $$

In the formula, *n* is the number of regions. This article was divided by provinces, autonomous regions, and municipalities in China, so n = 31*. X* is the cumulative percentage of the population in the corresponding area and*Y* is the percentage of medical resources serving the corresponding area. The value of Gini coefficient is between 0 and 1. If the value is closer to 0, which indicates the much fair the distribution of income or resources; if it is closer to 1, which means the more concentrated the income or resources, indicating the more unfair the distribution. According to international practice, 0.4 is usually used as a "guard line" for the gap in the allocation of medical and health resources. The Gini coefficient is <0.2, which means that the distribution of medical and health resources is highly fair; between 0.2 and 0.3, it is relatively fair; between 0.3 and 0.4, it is more reasonable; between 0.4 and 0.5 indicates a large gap; and >0.5 indicates a high degree of unfairness [12–14]

Theil index is mainly used to analyze the difference contribution of resource allocation between different regions, and it can also decompose the overall difference. In this study, the Theil index was used to compare the relative differences in the distribution of the 31 regions in China. The calculation formula for T is:

$$ {\displaystyle \begin{array}{l}\mathrm{T}=\sum {\mathrm{P}}_{\mathrm{i}}\times \log \left(\frac{\overline{R}}{R_i}\right)\\ {}\end{array}} $$

T is the Theil index, Pi is the proportion of the population in the area, $\overline{R}$ is a physician allocated by population (area) in the 31 regions, and R is the total population (area) in the 31 regions nationwide. The Theil index was calculated based on population (T_-population_) and area (T_-area_) in this study. The closer the T value is to 1, the worse the fairness is. T <0 indicates that the fairness in the province and city is higher than the overall fairness [15].

## Results

### Physician distribution in China

As shown in Table 1, the average number of physicians per 1,000 population in the 31 regions of China is 2.62. Jiangxi has the least number of physicians per 1,000 population, with less than 2 physicians per 1,000 population. Shanghai, Zhejiang, and Beijing have the highest number of physicians per 1,000 population, all exceeding 3 (Table 1). China's 31 regions have an average of 1.3 physicians per square kilometer. Physicians per square kilometer in Tibet, Qinghai, Xinjiang and a few other places have fewer medical physicians, showing less than 0.1 physician per square kilometer. Shanghai has the highest number of physicians per square kilometer, with more than 11 (Table 1).

**Table 1:** Basic distribution of physicians in 31 provincial administrative regions in China

State	Population (× 10^**3**^)	Total Area (× 10^**3**^ M^**2**^)	Total Physician (N)	Physician/10^**3**^ population(N)	Physician/10^**3**^ M^**2**^(N)
**Jiangxi**	4648	166,947	87,304	1.88	0.52
**Anhui**	6324	139,600	126,824	2.01	0.91
**Yunnan**	4830	394,000	99,669	2.06	0.25
**Guangxi**	4926	236,300	105,979	2.15	0.45
**Gansu**	2637	455,000	59,560	2.26	0.13
**Guizhou**	3600	176,100	81,475	2.26	0.46
**Fujian**	3941	121,400	91,110	2.31	0.75
**Heilongjiang**	3773	454,000	89,489	2.37	0.20
**Hainan**	934	35,000	22,289	2.39	0.64
**Tibet**	344	1,228,400	8322	2.42	0.01
**Guangdong**	11,346	179,800	276,361	2.44	1.54
**Henan**	9605	167,000	235,649	2.45	1.41
**Sichuan**	8341	485,000	204,956	2.46	0.42
**Chongqing**	3102	82,400	76,379	2.46	0.93
**Xinjiang**	2487	1,660,000	63,312	2.55	0.04
**Shaanxi**	3864	205,600	99,036	2.56	0.48
**Hubei**	5917	185,900	152,040	2.57	0.82
**Hunan**	6899	211,875	180,882	2.62	0.85
**Shanxi**	3718	156,000	99,490	2.68	0.64
**Qinghai**	603	722,000	16,153	2.68	0.02
**Liaoning**	4359	145,700	120,431	2.76	0.83
**Tianjin**	1560	11,305	43,105	2.76	3.81
**Hebei**	7556	190,000	211,387	2.80	1.11
**Ningxia**	688	66,400	19,435	2.82	0.29
**Jilin**	2704	187,400	77,108	2.85	0.41
**Shandong**	10,047	157,100	290,416	2.89	1.85
**Jiangsu**	8051	102,600	233,263	2.90	2.27
**Inner Mongolia**	2534	1,183,000	73,563	2.90	0.06
**Shanghai**	2424	6340.5	71,580	2.95	11.29
**Zhejiang**	5737	101,800	190,782	3.33	1.87
**Beijing**	2154	16,807.8	99,807	4.63	5.94
**Average**				2.62	1.33

### Equity of physician distribution in China

**Fig. 1:**
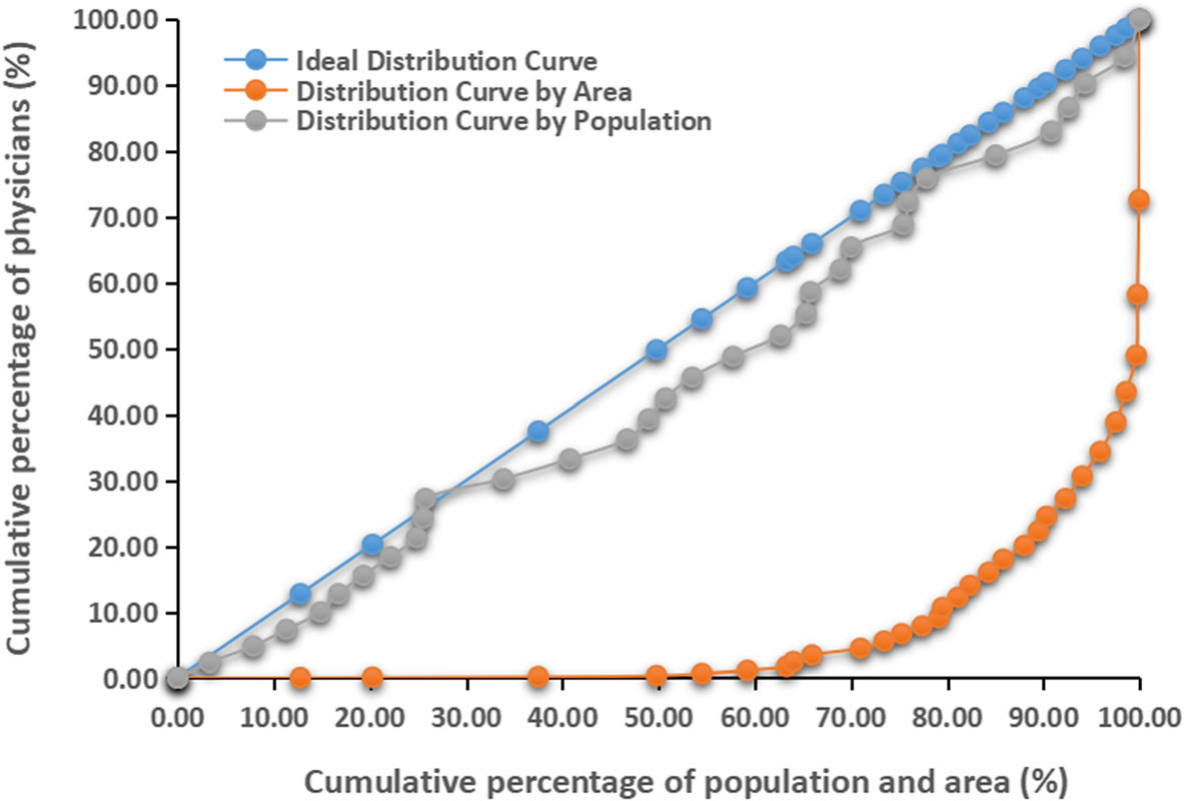
Lorentz curve of distribution of physicians by population and area in 31 provincial administrative regions of China

The Lorenz curve of the distribution of physicians by population in the 31 regions in China was generated based on the number of physicians per thousand population. The cumulative percentage of population in each region was taken as the X axis, and the cumulative percentage of physicians per 1,000 population was taken as the Y axis. On the same axis, the Lorenz curve of the distribution of medical physicians in the selected provinces and cities in China was generated according to the area of service (the service range/area involved in the practice of physicians), with the cumulative percentage of each province and city as the X axis and the cumulative percentage of physicians per square kilometer as the Y axis (Fig. 1).

According to the Lorenz curves of the distribution of medical physicians in the provinces and cities of China basing on population and service area, the Gini coefficients were calculated to be 0.0003 and 0.88, respectively. This means that according to the population, the distribution of Chinese medical physicians' resources is currently in a fairly fair state. However, according to the area of service, the distribution of Chinese PR is highly unfair.

### Fairness of physician distribution in China based on Theil index

As shown in Table 2, The overall distribution of physicians in the 31 regions in China is comparable basing on the calculation by population. Of which, Hunan Province is the most reasonable (Theil index is close to 0). The distribution of physicians in Anhui Province, Jiangxi and Yunnan is poor.

**Table 2:** Theil index of physicians by population and area in 31 provincial administrative regions in China

Province	T_**-population**_	T_**-area**_	State	T_**-population**_	T_**-area**_
**Jiangxi**	0.004797	0.007070	Hubei	0.000355	0.004054
**Anhui**	0.005212	0.002389	Hunan	0.000000	0.004277
**Yunnan**	0.003612	0.029697	Shanxi	− 0.000262	0.005146
**Guangxi**	0.003029	0.011548	Qinghai	−0.000042	0.136653
**Gansu**	0.001212	0.047712	Liaoning	−0.000706	0.003098
**Guizhou**	0.001655	0.008431	Tianjin	−0.000253	−0.000537
**Fujian**	0.001543	0.003136	Hebei	−0.001561	0.001549
**Heilongjiang**	0.001177	0.038788	Ningxia	−0.000157	0.004560
**Hainan**	0.000267	0.001154	Jilin	−0.000708	0.009945
**Tibet**	0.000085	0.270896	Shandong	−0.003065	− 0.002338
**Guangdong**	0.002511	−0.001189	Jiangsu	−0.002542	− 0.002473
**Henan**	0.002004	−0.000440	Inner Mongolia	−0.000800	0.165300
**Sichuan**	0.001634	0.025210	Shanghai	−0.000894	−0.000612
**Chongqing**	0.000608	0.001329	Zhejiang	−0.004278	−0.001564
**Xinjiang**	0.000209	0.262302	Beijing	−0.003814	−0.001134
**Shaanxi**	0.000278	0.009449			

T_-population_ = Theil index by population and T_-area_ = Theil index by area

The allocation of physicians by service area revealed that is higher in Shandong, Zhejiang, Guangdong, and Beijing than the national level. The distribution of the physicians in Henan Province is the most reasonable one, slightly better than the overall national level, while Tibet is very unfair, followed by Xinjiang and Inner Mongolia.

Overall, analysis of the distribution of general physicians by both population and service area showed that Beijing, Zhejiang, Shanghai, Jiangsu, Shandong, and Tianjin are higher than the overall level of 31 provincial administrative regions across the country (T_-population_ <0 and T_-area_ <0). The number of medical physicians is over-provisioned in Zhejiang, Shandong, Beijing and Jiangsu.

## Discussion

As data presented in this study, China had 2.62 practicing (assistant) physicians per 1,000 population by the end of 2018. This means that China has completed ahead of time the goal of 2.5 physicians per 1000 permanent residents by 2020 set by Chinese central government (Notice on Printing and Distributing the Outline of the National Medical and Health Service System Planning 2015-2020). However, physician resource is not evenly distributed and 11 of the 31 provinces have not reached the goal (2.5 medical physicians per 1,000 population) yet. This could be due to the long training period for medical students, and it is difficult to significantly increase the number of regional physicians in a short period of time. It is common for physicians in the U. S, Australia, and other high-income nations to practice medicine in both public hospitals and private clinics at the same time [16]. However, physicians in China are a unit member of hospitals, and their salary, welfare, and career development are closely related to the hospital. The lack of protection for multi-point practice greatly reduces doctors' enthusiasm for their practice. Therefore, it is important for policy makers in the health sector to formulate detailed implementation rules for physicians working at different sites, performance distribution systems, and insurance policies in accordance with the health needs of the people in the region and the wishes of the physician group. In addition, it is crucial to continuously improve and promote the model of physicians working in multiple places, and improve the accessibility of physicians.

This study showed that although the distribution of medical physicians by service area is highly unfair (G = 0.88), the co-efficiency by served population was 0.003, Our results of this study are consistent with the reports from other related studies, showing the fairness of physicians’ distribution according to population are much higher than the distribution basing on service area [17]. Since the allocation of medical resources should be guided by human needs, the accuracy and feasibility of the fairness of physicians’ distribution should be given more considered in future.

This study revealed that the fairness of medical physician distribution among the 31 provincial administrative regions is very high basing on served population. Although the fairness of quantity does not necessarily mean that the medical service level of physicians is the same, the fairness of quantity can allow people in different regions to get similar opportunity for medical services. Since the number of physicians in Zhejiang, Shandong, Beijing, and Jiangsu is over-provisioned, it may be attractive to more physicians from these provinces to the needed regions such as Anhui, Jiangxi and Yunnan by more incentive policy.

The distribution of physicians according to service area is highly unfair among the 31 regions in China. Tibet, Xinjiang, Inner Mongolia and other places are known to be vast and sparsely populated. The number of medical physicians per square kilometer in these areas is significantly less than that in Zhenjiang and Beijing. In addition, China's high-quality medical resources are mainly concentrated in large hospitals of eastern cities. The physicians at the community hospitals are obviously inferior to the tertiary hospitals in terms of education and experience [18, 19]. This makes it more difficult for residents in remote areas to seek medical services. Existing regional differences in physician allocation may be further exacerbated by evolving urbanization.

Today, the internet to conduct video consultations or health management of patients has been used to alleviate the shortage of medical resources in some high-income countries including the United States, the United Kingdom, and Japan [20, 21]. Internet medical services also started in China in 1990s, and online service has been developed and used in the areas of chronic disease management, maternal and child health, medical imaging, and medical education [22]. The Internet medical industry is gradually emerging in China and has been extended to the areas of chronic disease management, maternal and child health, medical imaging, and medical education. At present, it has developed to the online diagnosis and treatment stage represented by Internet hospitals [23]. The importance to strengthen the internet medical infrastructure in remote areas such as Tibet and Xinjiang is apparent. The use of the Internet for diagnosis and treatment can not only solve the problem of the highly unfair distribution of physicians by the service area and the difficulties of ordinary people in seeking medical treatment, but also promote the sinking of physicians' resources and make it easier for residents to obtain quality medical services. At the same time, the provision of medical services is greatly affected by public financial policies. Many low- and middle-income countries lack equal access to basic public health services due to lack of sustainable public financial support, and high rates of maternal and child mortality [24]. Therefore, the government also needs to promote the rational allocation of Chinese physicians' resources through a financing strategy that continuously improves basic public health equalization.

There are many statistical and quantitative research methods on the fairness evaluation of health resource allocation, such as Lorentz curve, Gini coefficient, Theil index, range method, difference index, concentration index method, etc. [25–27]. It is known that drawing the LC and calculating the G to judge the fairness is intuitive and easy to understand. However, the limitation is that it can only reflect the overall difference but not the fairness within the region. The Theil index can well reflect the contribution of intra-group gaps and inter-group gaps to the total gap, and is complementary to the Gini coefficient [28]. Since the limitation of individual method, more accurate assessment can be achieved with combined uses of more analysis methods. In this study, our analysis for the fairness of the physician's resource allocation in the 31 provincial administrative regions in China was conducted by calculating the Gini coefficient and Theil index. Thus, the findings of this study is more valid.

The distribution of physician resources is affected by many factors such as regional economic level and disease spectrum, and subjected to change over time. This study analyzed the fairness of the distribution of Chinese physicians basing on statistical data in 2019, which could provide the latest theoretical basis for the Chinese health department to formulate policies to allocate physician resources. In addition, the fairness in the distribution of physician resources is an important issue not just for China but also for many other countries. It would be beneficial for residents of any country, especially for the areas in the shortage of physician resources, to increase access to physicians by encouraging them to practice at multiple places and use the internet for medical service.

This study has some limitations. First of all, the research object of this article is the practicing (assistant) physicians in the 31 regions in China, and it cannot be distinguished whether the internal physician and other resource allocation of the 31 regions in China is fair. The future study needs to be conducted by focusing on specialized physician groups such as surgeons, pediatricians, obstetricians & gynecologists, and etc. for improved assessment of fairness distribution of physician resources. Secondly, the research results show that the distribution of Chinese physicians by service area is highly unfair. Whether this is directly related to the population density of various regions needs further analysis, such as Lorentz curve and different index methods.

## Conclusion

More and more researches have been devoted to the allocation of medical resources in recent year, which emphasizes the rational allocation of physician resources to be a focus of the medical industry. Our statistical analysis of the basic distribution of medical physicians revealed that the current number of physicians per 1,000 population in China has reached the staged development goals of China's health service system. The continuous growth in the number of physicians can further meet the medical and health needs of the people. This study showed that the fairness of Chinese physician resources is significantly higher by population than by service area, which substantiates the results of previous studies. Current development of physician practice in multiple sites, internet-based diagnosis and treatment are very encouraging and will greatly improve the current shortage of physicians in terms of population distribution and alleviate the problem of medical treatment in remote areas. In addition, future management on medical resources and services can be improved by strengthening the effective balance and coordination of high-quality medical resources and reducing the difference in physicians' quality among provinces and cities. And the assessment for fair distribution of medical physicians and other resources needs to be performed by using multiple analysis methods.

## Data Availability

The data for this study came from: a). China Health Statistics Yearbook 2019 (https://data.cnki.net/area/Yearbook/Single/N2020020200?z=D09) and b). China National Bureau of Statistics official website (http://www.stats.gov.cn/tjsj/ndsj/)

## Abbreviations

COVID-19: Coronavirus disease of 2019
PR: Physician resources
G: Gini coefficient
FA: Fairness assessment
LC: Lorenz curve
T: Theil index
T_-population_: Theil index based on population
T_-area_: Theil index based on area
